# Leishmanicidal Effect of Synthetic *trans*-Resveratrol Analogs

**DOI:** 10.1371/journal.pone.0141778

**Published:** 2015-10-30

**Authors:** Carlos Luan Alves Passos, Christian Ferreira, Deivid Costa Soares, Elvira Maria Saraiva

**Affiliations:** Instituto de Microbiologia Paulo de Góes, Universidade Federal do Rio de Janeiro, Rio de Janeiro, RJ, Brazil; UMR INSERM U866, FRANCE

## Abstract

**Background:**

Stilbene-based compounds show antitumoral, antioxidant, antihistaminic, anti-inflammatory and antimicrobial activities. Here, we evaluated the effect of the *trans*-resveratrol analogs, pterostilbene, piceatannol, polydatin and oxyresveratrol, against *Leishmania amazonensis*.

**Methodology/Principal Findings:**

Our results demonstrated a low murine macrophage cytotoxicity of all four analogs. Moreover, pterostilbene, piceatannol, polydatin and oxyresveratrol showed an anti-*L*. *amazonensis* activity with IC_50_ values of 18 μM, 65 μM, 95 μM and 65 μM for promastigotes, respectively. For intracellular amastigotes, the IC_50_ values of the analogs were 33.2 μM, 45 μM, 29 μM and 30.5 μM, respectively. Among the analogs assayed only piceatannol altered the cell cycle of the parasite, increasing 5-fold the cells in the Sub-G0 phase and decreasing 1.7-fold the cells in the G0-G1 phase. Piceatannol also changed the parasite mitochondrial membrane potential (ΔΨm) and increased the number of annexin-V positive promastigotes, which suggests incidental death.

**Conclusion/Significance:**

Among the analogs tested, piceatannol, which is a metabolite of resveratrol, was the more promising candidate for future studies regarding treatment of leishmaniasis.

## Introduction

Leishmaniasis is a public health problem that affects 98 countries on 5 continents, and approximately 1.1 to 1.7 million cases of leishmaniasis occurs each year [1]. Pentavalent antimonials are the first-line drugs for treating leishmaniasis; amphotericin B, pentamidine, and paramomycin, are secondary options for the treatment of resistant cases [2, 3]. All of these drugs have problems that limit their use, such as side effects, the induction of parasite resistance, in-patient administration and high costs [4]. Miltefosine is the first approved oral treatment for leishmaniasis in India; however, it has low efficacy against cutaneous leishmaniasis and is teratogenic [5, 6]. Thus, the search for new drugs for this neglected disease is needed and has been stimulated by the Drugs for Neglected Diseases initiative (DNDi).

Recently, we demonstrated that the stilbene resveratrol kills *Leishmania amazonensis* through incidental death [7]. Importantly, we also demonstrated that resveratrol is active against amastigotes and synergizes with amphotericin B.

Studies of chemical structures associated with biological properties of analogs will aid in the search for new drugs for disease treatment by aiding in the development of more active drugs and drugs that are less toxic to the host.

Resveratrol has different analogs, and several biological effects of these analogs have already been described: piceatannol (*trans*-3,4,3',5'-tetrahydroxystilbene) inhibits tyrosine kinase activity in lymphoid malignancies [8, 9]; pterostilbene (*trans*-3,5-dimethoxy-4-hydroxystilbene) activates caspases 3/7 and has antioxidant, anti-inflammatory and anticarcinogenic properties [10, 11]; polydatin (resveratrol-3-O-β-mono-D-glucoside) has anti-inflammatory and antioxidant activities, decreases IL-17 production in activated human peripheral blood mononuclear cells [12–14], protects cardio-myocytes and antagonizes platelet aggregation, thrombosis, and atherosclerosis [15]; oxyresveratrol (*trans*-2,3′,4,5′-tetrahydroxystilbene) inhibits nitric oxide (NO) and decreases prostaglandin E2 (PGE2) production, inhibits the activation of NF-κB in macrophages and consistently reduces edema induced by carrageenan in mice [16].

**Fig 1:**
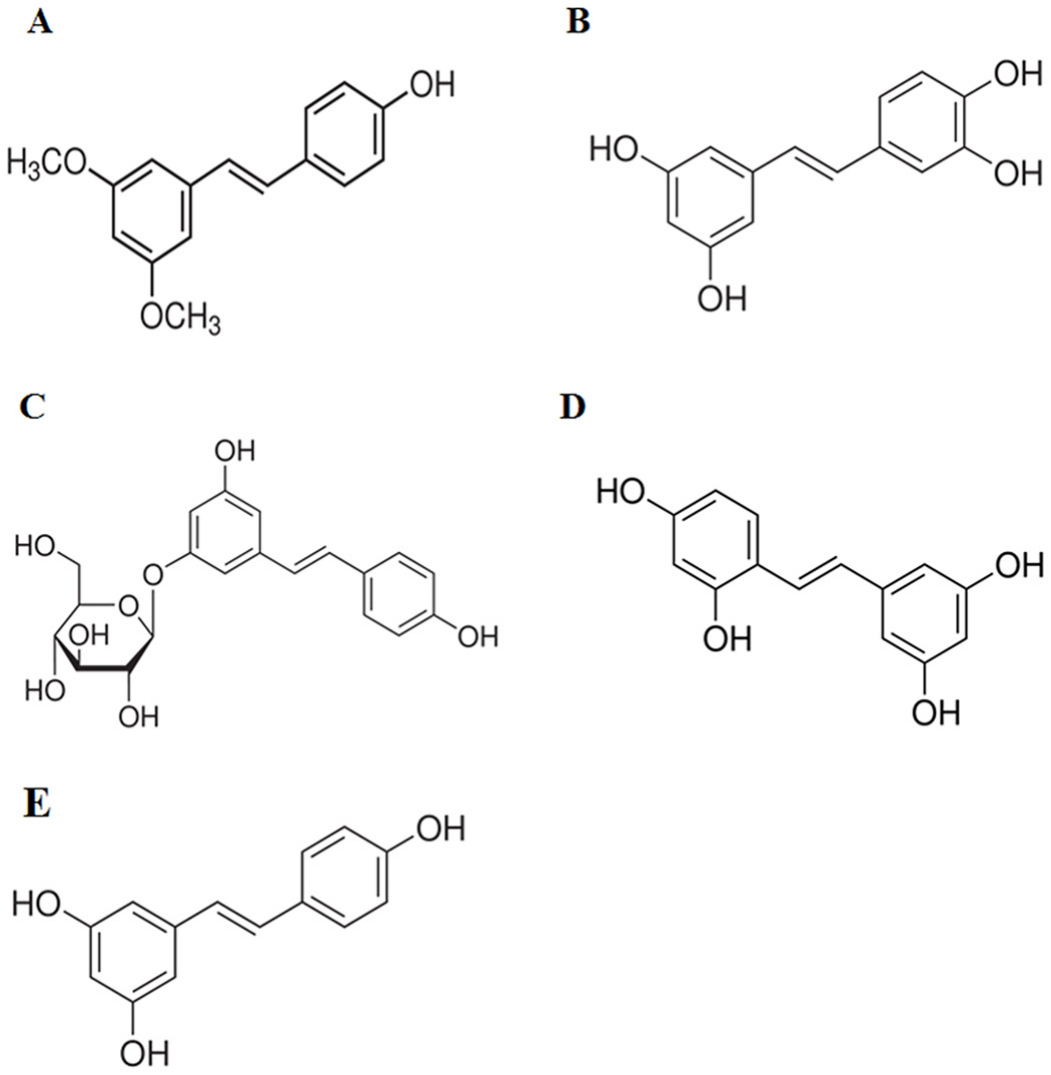
Chemical structure of (A) Pterostilbene (B) Piceatannol, (C) Polydatin, (D) Oxyresveratrol, (E) *trans*-Resveratrol.

Here, we report the activity of these four resveratrol analogs (pterostilbene, piceatannol, polydatin and oxyresveratrol (Fig 1) against *Leishmania amazonensis*, which, in humans, may cause cutaneous leishmaniasis, a severe anergic diffuse cutaneous leishmaniasis and the visceral form of this disease [17].

## Methods

### Ethics Statement

BALB/c mice (8–10 weeks) obtained from Federal Fluminense University animal facility were housed at Federal University of Rio de Janeiro in temperature-controlled room (23 ± 1°C), with a 12/12 h light/dark cycle, and water and food *ad libitum*. Euthanasia was carried out in a saturated atmosphere of isoflurane, and all efforts were made to minimize animal suffering. All experiments with animals were performed in strict accordance with the Brazilian animal protection law (Lei Arouca number 11.794/08) of the National Council for the Control of Animal Experimentation (CONCEA, Brazil). This study protocol was approved by the Committee for Animal Use of the Universidade Federal do Rio de Janeiro (Permit Number: IMPPG 024).

### Drugs

Pterostilbene (*trans*-3,5-dimethoxy-4-hydroxystilbene), piceatannol (*trans*-3,4,3',5'-tetrahydroxystilbene), polydatin (resveratrol-3-O-b-mono- D-glucoside), oxyresveratrol (*trans*-2,3′,4,5′-tetrahydroxystilbene) and DMSO were purchased from Sigma. Miltefosine was donated by Dr. Norton Heise (Universidade Federal do Rio de Janeiro, RJ, Brazil).

### Parasite Culture

*L*. *amazonensis* (WHOM/BR/75/Josefa) promastigotes were cultured at 26°C in Schneider insect medium (Sigma), 10% fetal calf serum (Gibco-BRL, MD, US.) and 20 μg/mL of gentamycin (Schering-Plough, Rio de Janeiro, Brazil).

### Anti-promastigote Activity

The leishmanicidal properties of the analogs were evaluated by measuring promastigotes’ mitochondrial activity using the XTT method with 2,3-bis-[2-methoxy-4-nitro-5-sulphophenyl]-2H-tetrazolium-5-carboxanilida, Sigma) as described previously [18]. Briefly, stationary-phase promastigotes were treated with different concentrations of pterostilbene, piceatannol, polydatin and oxyresveratrol for 48 h at 26°C and were then incubated with XTT activated with phenazine methosulfate (PMS, Sigma) for 3 h. Sodium azide (10 μM) was used as a control, and the reaction product was read at 450 nm.

### Anti-amastigote Activity

Mice peritoneal macrophages obtained after stimulation with thioglycolate for 3 days were harvested in RPMI 1640 medium (LGC Biotecnologia, São Paulo, Brazil), 20 μg/mL of gentamycin and were plated onto 13 mm^2^ coverslips inside 24-well plates for adherence for 2 h at 37°C, 5% CO_2_. Non-adherent cells were removed, and the macrophages were incubated overnight in RPMI supplemented with 10% FCS, as described above. Adhered macrophages were infected with *L*. *amazonensis* promastigotes (stationary growth phase) at a 10:1 parasite/macrophage ratio and incubated for 1 h at 34°C, 5% CO_2_. Free parasites were washed out with 0.01 M phosphate buffered saline (PBS), and the cultures maintained for 24 h at 37°C, 5% CO_2_. Infected macrophage cultures were treated with different concentrations of the analogs for an additional 24 h at 37°C, 5% CO_2_. Monolayers were then washed with PBS at 37°C, fixed in methanol, and stained with Giemsa. The number of amastigotes and the percentage of infected macrophages were determined by counting at least 200 cells in duplicate cultures. Endocytic indices were obtained by multiplying the percentage of infected macrophages by the mean number of amastigotes per infected macrophage. The results were expressed as the percentage of surviving macrophages based on endocytic indices of treated and untreated macrophages as reported previously [19].

### Cytotoxicity for host macrophages

Mice peritoneal macrophages adhered to 96-well plates were treated with pterostilbene, piceatannol, polydatin or oxyresveratrol for 24 h, and cell viability was determined using 1 mg/mL XTT (2,3-Bis[2-Methoxy-4-nitro-5-sulfophenyl]-2H-tetrazolium-5-carboxinilide inner salt, Sigma), 200 μM PMS (Phenazine Methosulfate). After 3 h of incubation, the reaction product was read at 450 nm. The results are expressed in percentage of viable cells compared to untreated control [20, 21].

### Selectivity Index Calculation

The selectivity index was calculated as the ratio of murine peritoneal macrophages CC_50_ by *L*. *amazonensis* intracellular amastigotes IC_50_.

### Nitric Oxide Production

Thioglycolate-stimulated peritoneal macrophages were obtained as described above (10^6^ cells/well in 24-well plate) and were activated with 1 μg/ml LPS (Sigma-Aldrich) or left untreated. After incubating the cells for 24 h at 37°C, 5% CO_2_, they were treated with 33.2 μM pterostilbene, 45 μM piceatannol, 29 μM polydatin and 30.5 μM oxyresveratrol. The nitrite concentrations in the culture supernatants were determined using the Griess method. The reaction was read at 540 nm, and the concentration of NO_2_^-^ determined using a standard curve of sodium nitrite. The results are expressed as micromolar concentrations of nitrite [22].

### Nitric oxide-trapping capacity

A cell-free system with an NO donor was used to test the capacity of resveratrol analogs to trap NO. SNAP (s-nitroso n-acetyl DL-penicillamine, Sigma) liberates nitric oxide in solution, which is then transformed to nitrite in the medium. The addition of a NO scavenger to the SNAP solution results in nitrite decay in the supernatant. Using this protocol, 33.2 μM pterostilbene, 45 μM piceatannol, 29 μM polydatin and 30.5 μM oxyresveratrol were incubated with 1 mM SNAP. One milimolar rutin (Sigma), a known NO scavenger, was used as a positive control for this assay. After 6 h of incubation, the nitrite concentration was determined using the Griess method. The results are expressed as the μM concentration of nitrite calculated in comparison with the sodium nitrite standard curve [18].

### Detection of Reactive Oxygen Species (ROS)

Mice peritoneal macrophages were adhered to 96-well opaque culture plates and then infected or not with *L*. *amazonensis* promastigotes. Twenty-four hours post-infection, they were treated with pterostilbene, piceatannol, polydatin and oxyresveratrol at their respective IC_50_ concentrations and were stimulated or not with 1 μg/mL phorbol 12-myristate 13-acetate (PMA, Sigma). The cells were then stained with 50 μM dihydrorhodamine 123 (DHR 123, Life Technologies), and ROS was measured immediately using 500/526 nm excitation/emission wavelengths.

### Cell Cycle Analysis

Promastigotes were incubated in Schneider's complete medium, with or without 17.7 μM pterostilbene, 65 μM piceatannol, 95.5 μM polydatin, 65 μM oxyresveratrol and 1% DMSO for 48 h. The cells were washed with PBS and fixed in 70% (v/v) ice-cold methanol/PBS for at least 1 h at 4°C. The fixed cells were washed once with PBS and then incubated in PBS supplemented with 10 μg/mL propidium iodide (PI) and 20 μg/mL RNAse at 37°C, 45 min, as reported [23]. For each sample, 10000 events were collected on a BD FACScalibur (Becton and Dickson) and analyzed using CellQuest software.

### Measurement of mitochondrial membrane potential (ΔΨm)

Change in ΔΨm was measured using the mitochondria staining kit (Sigma-Aldrich). Promastigotes were treated or not with 17.7 μM pterostilbene, 65 μM piceatannol, 95.5 μM polydatin and 65 μM oxyresveratrol for 48 h and then incubated with JC-1 (5 μg/mL) staining solution (prepared according to the manufacturer’s instructions) for 20 min at 37°C. ΔΨm was measured in 96-well opaque plates using 490/530 nm excitation/emission wavelengths for JC-1 monomers and 525/590 nm excitation/emission wavelengths for J-aggregates in a SpectraMax Paradigm (Molecular Devices), as described previously [18].

### Annexin V binding assay

Double staining for annexin V-fluorescein isothiocyanate (FITC) and PI was performed with the Annexin-V apoptosis detection kit (Molecular Probes). Promastigotes were treated or not with different concentrations of piceatannol for 48 h. They were then washed twice in cold annexin V-buffer and centrifuged at 2760 x g for 10 min. Pellets were resuspended in 20 μL of annexin V FITC, and after 15 min of incubation in the dark, 480 μL of annexin V-buffer was added according to the manufacturer instructions. Annexin V-FITC labeling was recorded on a BD FACScalibur (Becton and Dickson) and analyzed using CellQuest software.

### *In Silico* Analyses

*In silico* theoretical analyses were carried out as proposed by Pinheiro *et al*. [24], and different descriptors, such as octanol/water coefficient partition (ClogP), molecular weight (MW), molecular volume (MV), number of hydrogen bonds donated (HBD) and number of hydrogen bonds accepted (HBA), were evaluated using OSIRIS Property Explorer (http://www.organic-chemistry.org/prog/peo) and admetSAR (http://lmmd.ecust.edu.cn:8000) methods.

### Statistical Analysis

Data were analyzed using Student's t-test when comparing two groups or one-way ANOVA for more than two groups using the software GraphPad Prism. P values of less than 0.05 were considered significant.

## Results

### Anti-promastigote Activity

Initially, the activity of *trans*-resveratrol analogs on *Leishmania amazonensis* promastigotes survival was assayed (Table 1). Our results demonstrated an anti-leishmanial activity of these analogs with IC_50_ values of 18, 65, 95.5 and 65 μM for pterostilbene, piceatannol, polydatin and oxyresveratrol, respectively, after 48 h of treatment. The IC_50_ of Amphotericin B used as a control was 0.1 μM (Table 1).

**Table 1:** *In vitro* anti-leishmanial activity and cytotoxicity results for *trans*-Resveratrol Analogs.

	IC_50_	IC_50_	CC_50_	
Drug	Promastigotes	Intracellular Amastigotes	Peritoneal Macrophages	SI *
**Pterostilbene**	17.7 μM	33.2 μM	181.0 μM	5.4
**Piceatannol**	65.0 μM	45.0 μM	>400.0 μM	>8.9
**Polydatin**	95.5 μM	29.0 μM	150.0 μM	5.2
**Oxyresveratrol**	65.0 μM	30.5 μM	128.0 μM	4.2
**Amphotericin B**	0.1 μM	8.8 nM	>5.0 μM	n.d.

* The selectivity index (SI) was calculated as the ratio of CC_50_ on murine peritoneal macrophages to IC_50_ on *L*. *amazonensis* intracellular amastigotes.

n.d.–not determined.

### Host cell viability assays

To test the safety of *trans*-resveratrol analogs on host cells, we evaluated the dehydrogenases activity of macrophages using the XTT method. Our results indicated that the cytotoxic concentration for 50% of the population (CC_50_) was 181, >400, 150, and 128 μM for pterostilbene, piceatannol, polydatin and oxyresveratrol, respectively (Table 1). The CC_50_ of Amphotericin B was > 5 μM.

### Anti-amastigote activity

Next, we tested the effect of *trans*-resveratrol analogs on amastigotes, which are the parasite forms present in the mammalian host and are responsible for the infection. The anti-amastigote activity was tested after 24 h of treatment, and our results showed that the IC_50_ value of pterostilbene, piceatannol, polydatin and oxyresveratrol was calculated as 33.2 μM, 45 μM, 29 μM and 30.5 μM, respectively (Table 1). The IC_50_ of Amphotericin B was 8.8 μM.

### Nitric Oxide (NO) Production and Nitric Oxide-trapping Capacity

**Fig 2:**
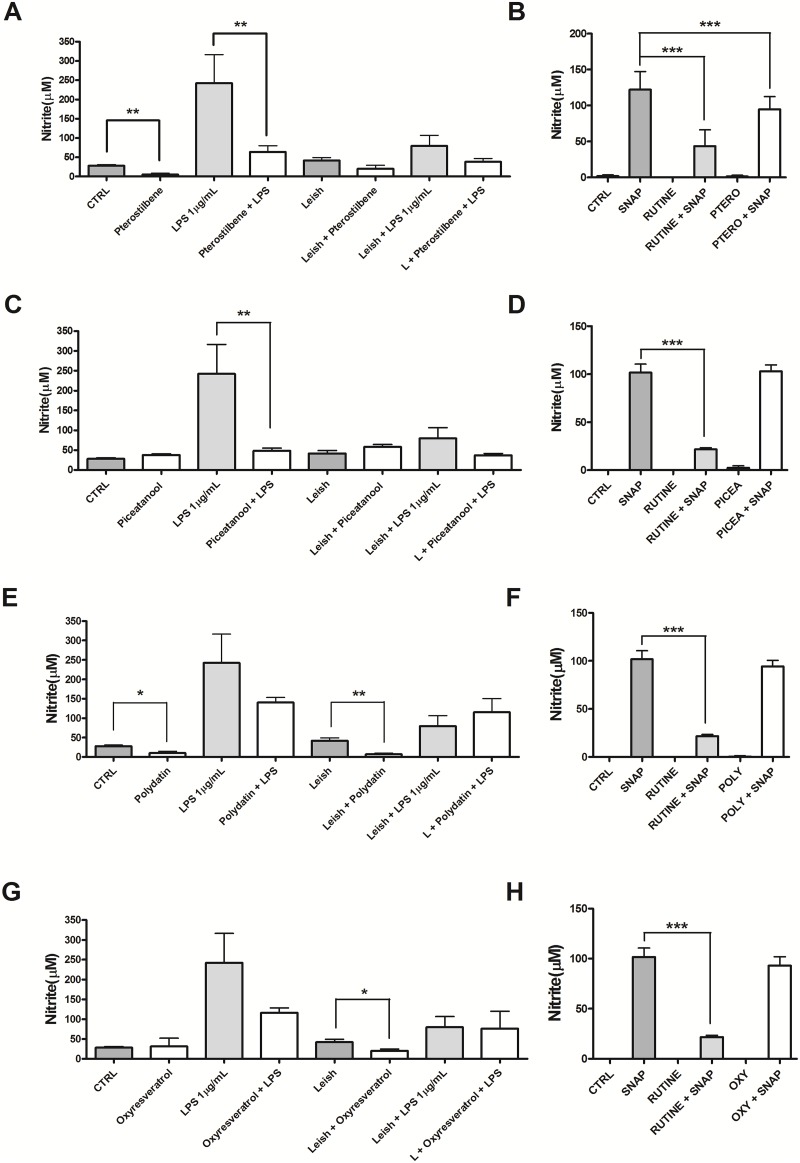
Effects of *trans*-Resveratrol on nitric oxide (NO) production by murine peritoneal macrophages. Uninfected macrophages (10^5^) and macrophages infected with *Leishmania amazonensis* at a 10:1 ratio stimulated with LPS or left unstimulated were incubated in the presence or absence of 33.2 μM pterostilbene (**A**), 45 μM piceatannol (**C**), 29 μM polydatin (**E**) and 30.5 μM oxyresveratrol (**G**). NO production was evaluated after 48h of treatment by the Griess method. The results of two independent experiments performed in duplicate are shown as the mean ± SEM nitrite concentration. * P <0.05, ** P <0.001, *** P<0.0001. Scavenger effect analysis was performed in a cell-free system by incubating SNAP solution (1 mM) as the NO donor with the same concentrations of *trans*-resveratrol used in NO (**B**, **D**, **F**, **H**). Rutin (1 mM), an NO scavenger, was used as positive control, and RPMI medium served as negative control. Nitrite levels were determined by the Griess method. The data represent the mean ± SEM of three independent experiments. * P <0.05, ** P <0.01, *** P <0.0001.

NO is recognized as an important mediator of *Leishmania* death, therefore we investigated whether *trans*-Resveratrol analogs were able to modulate NO production by macrophages. Our results showed that pterostilbene decreased the NO production of uninfected macrophages stimulated or not with LPS by 5.3- and 3.8-fold, respectively (Fig 2A). Piceatannol decreased the NO production only in the uninfected macrophages stimulated with LPS by 5.0-fold (Fig 2C). The treatment of uninfected and unstimulated macrophages with polydatin reduced NO production by 2.8-fold, and polydatin or oxyresveratrol treatments decreased the NO production of infected macrophages by 6.1- and 2.0-fold, respectively (Fig 2E and 2G).

We used a cell-free system to rule out a possible NO scavenging effect of the *trans*-resveratrol analogs. Thus, s-nitroso n-acetyl-DL-penicillamine (SNAP), a NO donor, was incubated in the presence or absence of pterostilbene, piceatannol, polydatin and oxyresveratrol. As a positive control, rutin, a NO scavenger, was added to the SNAP solution, and it decreased NO levels by 72%. Addition of piceatannol, polydatin or oxyresveratrol at 45 μM, 29 μM and 30.5 μM, respectively, did not reduce the levels of NO, indicating that the decrease in NO production that resulted from the treatment with these compounds was not due to a scavenging effect of the drugs (Fig 2D, 2F and 2H). However, 33.2 μM pterostilbene decreased NO levels by 22%, indicating that this analog has a NO scavenging effect (Fig 2B).

### Detection of Reactive Oxygen Species

**Fig 3:**
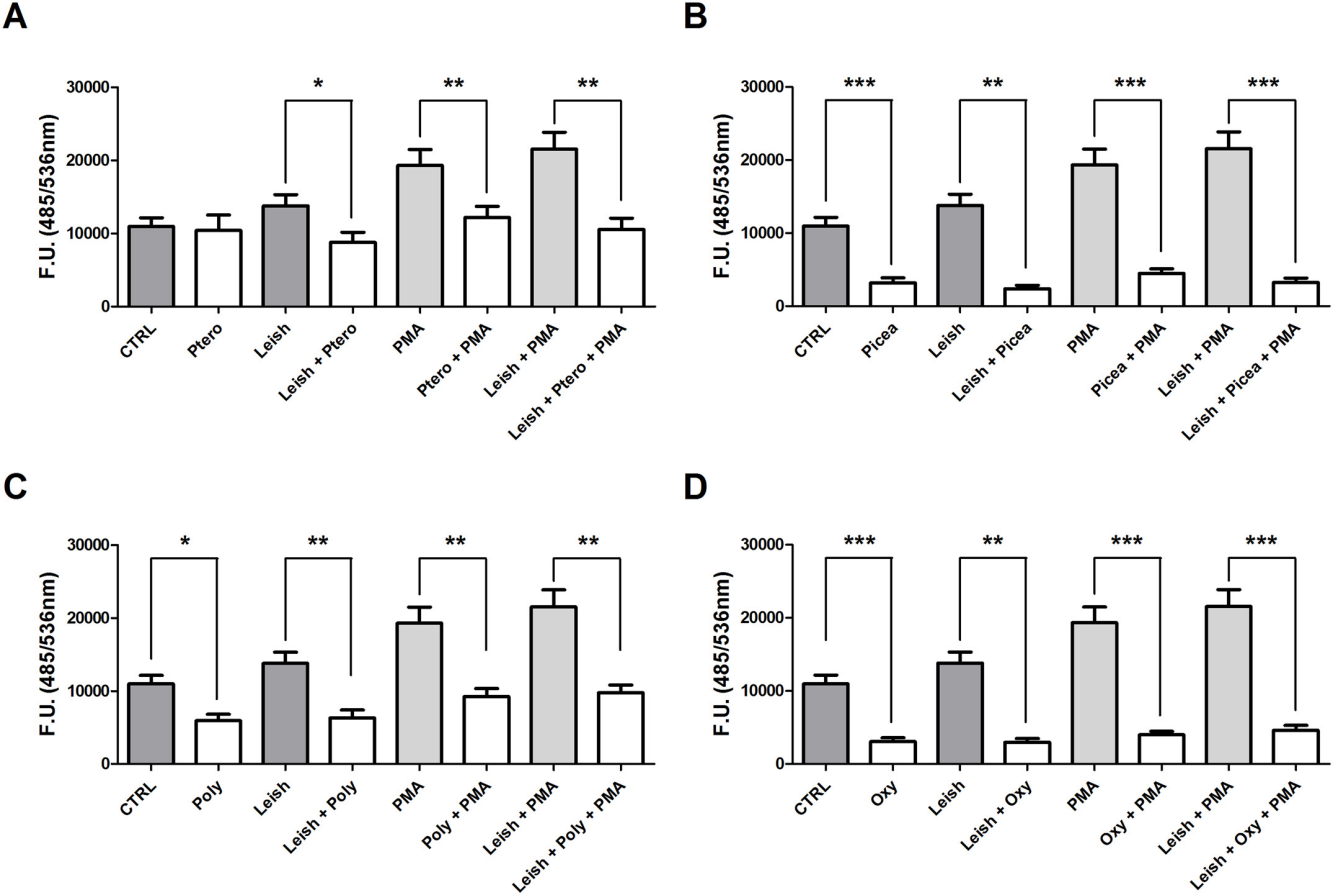
Effects of trans-Resveratrol on ROS production by murine peritoneal macrophages. Uninfected macrophages (10^5^) and macrophages infected with *Leishmania amazonensis* at a 10:1 ratio stimulated or not with PMA were incubated in the presence or absence of 33.2 μM pterostilbene (**A**), 45 μM piceatannol (**C**), 29 μM polydatin (**E**) or 30.5 μM oxyresveratrol (**G**) for 30 minutes. The data represent the mean ± SEM of three independent experiments. * P <0.05, ** P <0.01, *** P <0.0001.

Treatment of macrophages with pterostilbene decreased ROS levels in *L*. *amazonensis* infected-macrophages by 1.6-fold, and the stimulation of infected and uninfected macrophages with 1 μg/mL PMA decreased ROS levels by 1.6- and 2.0-fold, respectively (Fig 3A). For uninfected macrophages, piceatannol, polydatin and oxyresveratrol reduced ROS levels by 3.4-, 1.8- and 3.6-fold, respectively, and for infected macrophages, they reduced ROS levels by 5.8-, 2.1- and 4.6-fold, respectively (Fig 3B, 3C and 3D). For uninfected macrophages stimulated with PMA, piceatannol, polydatin and oxyresveratrol reduced ROS levels by 4.3-, 2.0-, and 4.8-fold, respectively, and for infected and PMA-stimulated macrophages, they reduced ROS levels by 6.6-, 2.2- and 4.7-fold, respectively (Fig 3B, 3C and 3D).

### Cell Cycle

**Fig 4:**
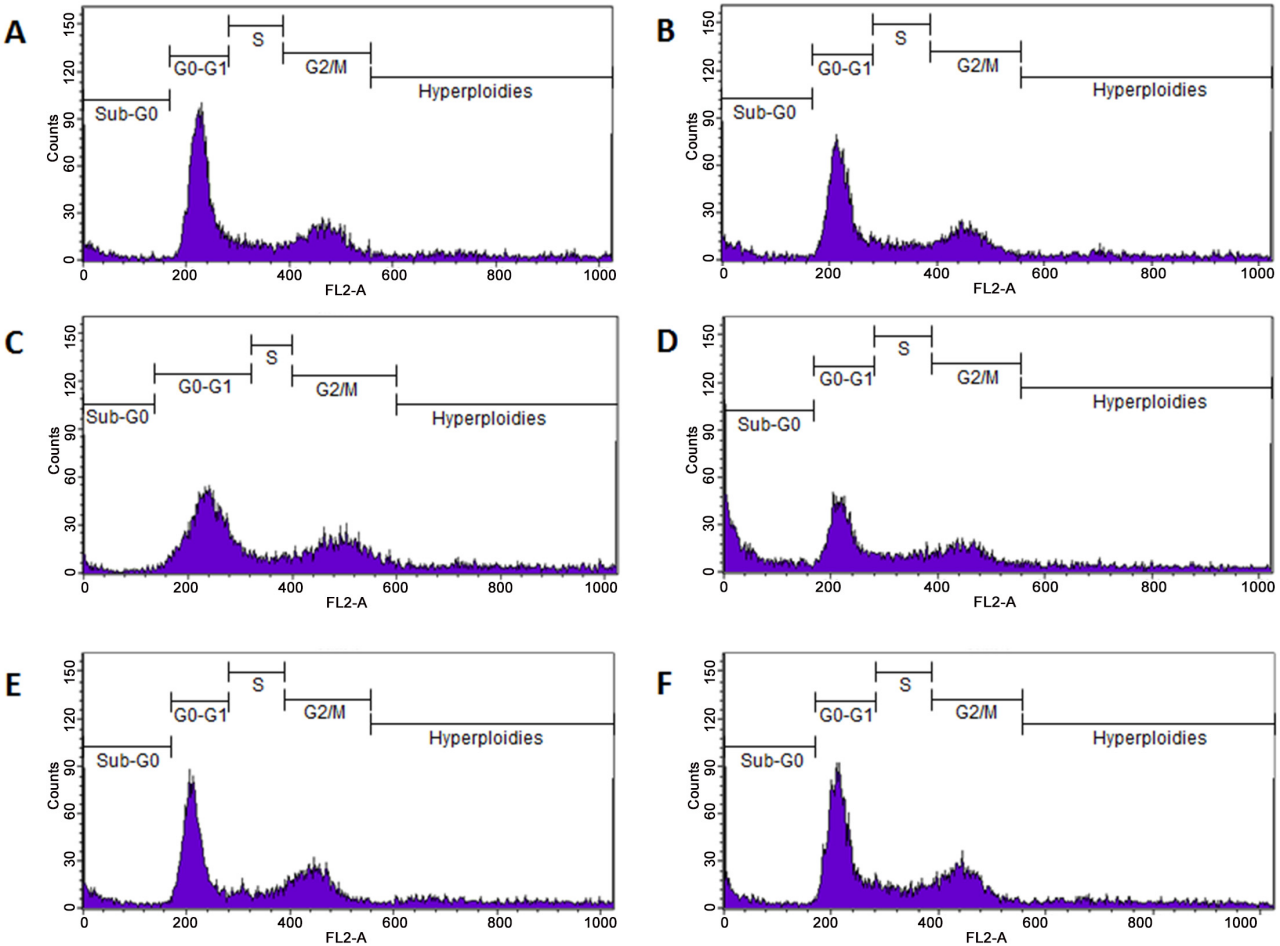
Evaluation of cell cycle through analysis of DNA content of *L*.*amazonensis*. Promastigotes were treated or not with *trans*-resveratrol analogs and cell cycle evaluated after PI staining by flow cytometry analysis. Untreated parasites control (**A**), 1% DMSO (**B**), 17.7 μM pterostilbene (**C**), 65 μM Piceatannol (**D**), 95.4 μM Polydatin (**E**) and 65 μM Oxyresveratrol (**F**). Representative plots of at least three independent experiments with similar results.

Previous results demonstrated that resveratrol treatment of *L*. *amazonensis* affected the parasite cell cycle [7], thus, we tested this property of the resveratrol analogs (Fig 4). Untreated promastigotes (Fig 4A), or treated during 48 h with 1% DMSO (Fig 4B), 17.7 μM pterostilbene (Fig 4C), 65 μM piceatannol (Fig 4D), 95.4 μM polydatin (Fig 4E) or 65 μM oxyresveratrol (Fig 4F), were labeled with propidium iodide in cell cycle solution and analyzed via flow cytometry. Our results showed that 65 μM piceatannol affected the division pattern of the promastigotes by increasing the cells in Sub-G0 phase 5-fold and decreasing the cells in G0-G1 phase 1.7-fold (Table 2). Pterostilbene, polydatin and oxyresveratrol did not alter the cell cycle of the parasite (Table 1).

**Table 2:** Analysis of *Leishmania amazonensis* promastigotes cell cycle.

TREATMENT	% CELLS IN DIFFERENT STAGES OF THE CELL CYCLE #				
	Sub-G0	G0/G1	S	G2/M	Hyperploidies
**CONTROL**	6.437 ± 2.586	37.735 ± 1.501	8.278 ± 0.315	22.610 ± 2.191	25.245 ± 2.588
**DMSO 1%**	7.633 ± 2.597	36.977 ± 0.984	8.620 ± 0.327	22.375 ± 1.809	24.687 ± 0.665
**PTEROSTILBENE 17.7 μM**	4.423 ± 0.216	42.380 ± 1.006	7.823 ± 0.915	24.713 ± 1.331	20.873 ± 0.309
**PICEATANNOL 65 μM**	30.990 ± 1.544***	21.890 ± 0.840***	8.167 ± 0.522	14.273 ± 0.966	24.947 ± 0.801
**POLYDATIN 95.4 μM**	13.250 ± 4.747	32.040 ± 2.699	8.698 ± 0.946	20.090 ± 2.661	26.233 ± 1.273
**OXYRESVERATROL 65 μM**	10.413 ± 3.895	34.380 ± 2.300	9.570 ± 0.444	21.073 ± 2.386	24.862 ± 2.500

^#^ Results are the mean ± SEM of at least three independent experiments.

*** P <0.001, in relation to control.

### Annexin-V Binding Assay

**Fig 5:**
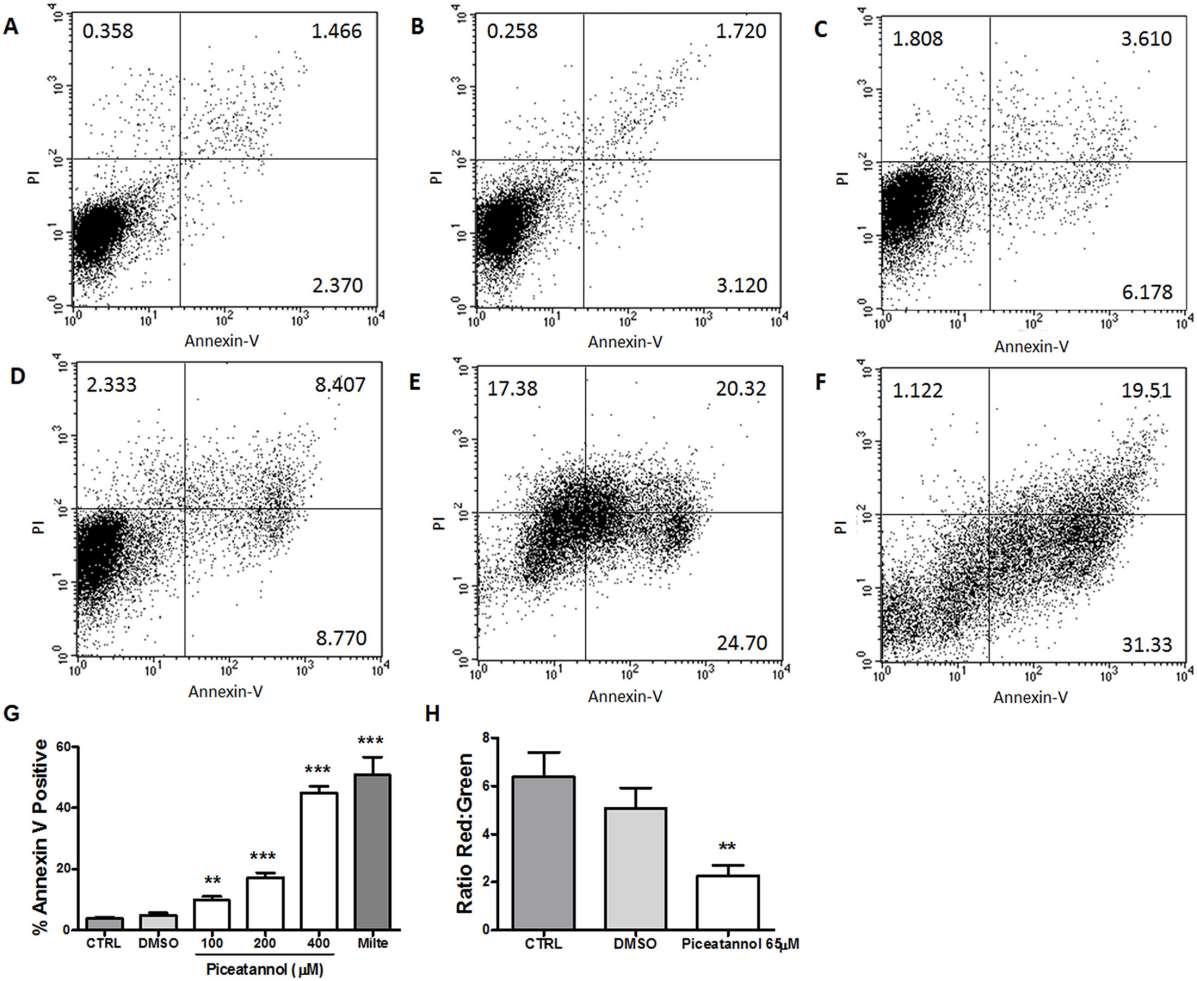
Incidental death of *L*. *amazonensis* parasites treated with Piceatannol. Promastigotes were treated or not with piceatannol and the incidental death measured by the expression of Annexin V. Untreated parasites control (**A**), 1% DMSO (**B**), parasites treated with piceatannol at 100 μM (**C**), 200 μM (**D**), 400 μM (**E**) and 30 μM miltefosine (**F**) are shown as a representative result. The percentage of Annexin-V positive promastigotes, treated as above, shown as mean ± SEM of three independent experiments (**G,** bars represent Annexin^+^ plus Annexin^+^/PI^+^ cells). *Leishmania* mitochondrial activity. Promastigotes were cultured 48h in the presence or absence of 65 μM piceatannol and 1% DMSO (vehicle). The mitochondrial membrane potential (ΔΨm) was evaluated using JC-1 (**H**). Results expressed as red/green fluorescence ratios, represent the mean ± SEM of 3 independent experiments. **P<0.001, *** P <0.0001, compared to control. Milte = Miltefosine.

The annexin V assay evaluated the potential induction of incidental death in promastigotes by piceatannol (Fig 5). Our results showed that piceatannol induced a dose-dependent increase in annexin-V labeling (Fig 5C–5E and 5G), and 100 μM piceatannol increased the percentage of annexin-V positive promastigotes by 2.5-fold in relation to untreated control (Fig 5G). Moreover, piceatannol induced a dose-dependent increase in PI labeling (Fig 5C–5E). Miltefosine, used as a positive control, increased the annexin-V binding to promastigotes by 13.2-fold (Fig 5F and 5G).

### Parasite Mitochondrial Alterations

*Leishmania* is a single mitochondrion parasite and this organelle is involved in apoptotic cell death. In order to address the parasite mitochondrial toxicity, reduction of the mitochondrial membrane potential (ΔΨm) was assessed in piceatannol-treated promastigotes using the JC-1 assay (Fig 5). Our results showed a reduction of 2.8-fold in the mitochondrial membrane potential in parasites treated with 65 μM piceatannol (Fig 5H). Treatment with 1% DMSO did not change the mitochondrial membrane potential.

### *In Silico* Evaluations

**Fig 6:**
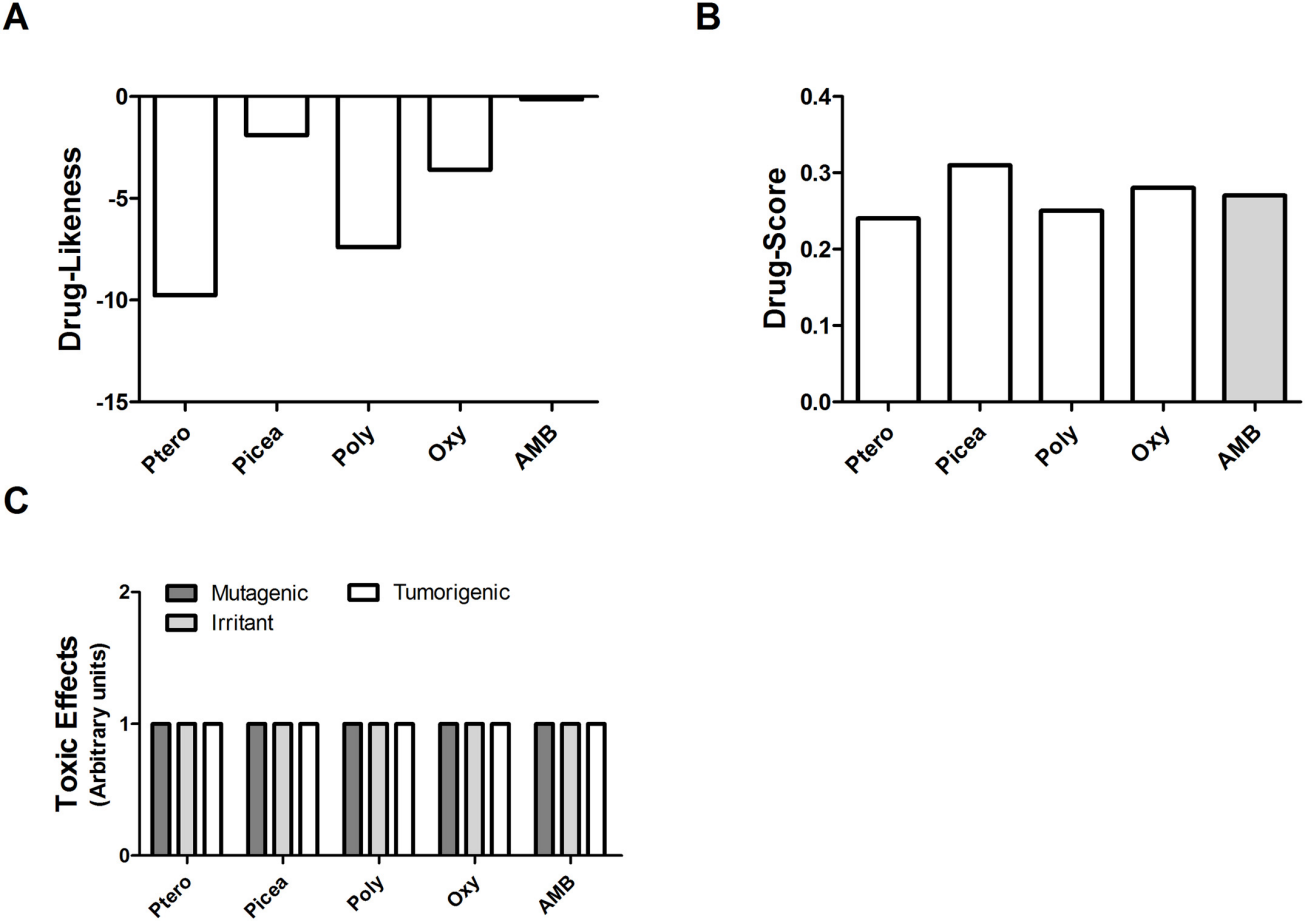
*In silico* evaluations of Pterostilbene, Piceatannol, Polydatin, Oxyresveratrol and Amphotericin B. (**A**) Comparative evaluation of drug-likeness profile; (**B**) drug-score; and (**C**) toxicity profile obtained by *in silico* evaluation using Osiris^®^ software.

The rate of drug-likeness indicates whether a compound has certain essential features observed in most of the drugs on the market. Thus, this index shows the potential of a certain compound as a drug candidate. Potential drug-likeness of the *trans*-resveratrol analogs was calculated, and AMB was used as a control because it is a drug widely used for leishmaniasis treatment. Among the analogs, only piceatannol showed a potential drug-likeness to AMB (Fig 6A).

The Drug-Score index is a parameter that combines characteristics of the compound (ClogP, solubility, theoretical toxicity, Drug-Likeness and molecular weight) and is calculated using Osiris software, which evaluates whether a certain compound might be a future drug candidate. According to established criteria, values closer to 1 indicate a higher probability of a compound being a future drug candidate. The *trans*-resveratrol analog that had the best drug-score was piceatannol (0.31). Pterostilbene, polydatin and oxyresveratrol had drug-score values of 0.24, 0.25 and 0.28, respectively. AMB had a drug-score value of 0.27 (Fig 6B).

The prediction of toxicological risks (mutagenic, tumorigenic, irritant and toxic risk in reproduction) of these compounds was also analyzed. All analogs showed no tumorigenic, irritant or mutagenic effects. AMB did not show any of the toxic effects evaluated (Fig 6C).

The results presented in Table 3 showed that there was no violation of the Lipinski’s rules [25], which evaluate physico-chemical parameters, suggesting the importance of the resveratrol analogs as drug candidates with good oral bioavailability.

**Table 3:** Lipinski´s rule of five of *trans*-Resveratrol Analogs and Amphotericin B.

Drug	CLogP	HBA	HBD	MW
**PTEROSTILBENE**	3.38	3	1	256.30
**PICEATANNOL**	2.48	4	4	244.24
**POLYDATIN**	0.84	8	6	390.38
**OXYRESVERATROL**	2.48	4	4	244.24
**AMB**	11.88	17	12	924.08

ClogP—octanol/water coefficiente partition; MW—molecular weight; MV—molecular volume;

HBD—number of hydrogen bonds donated; HBA—number of hydrogen bond accepted.

## Discussion

Stilbenes are natural products with low molecular weights isolated from several plant species, and resveratrol is by far the most studied among them, with several of its biological effects having already been described [26, 27]. Among its effects, we recently described the leishmanicidal activity of resveratrol against *L*. *amazonensis* [7]. Here, we describe the leishmanicidal effect of four *trans*-resveratrol analogs: pterostilbene, piceatannol, polydatin and oxyresveratrol. Our study showed, for the first time, that the analogs polydatin and oxyresveratrol are active against *L*. *amazonensis* promastigotes and amastigotes. Considering the CC_50_ determined using the XTT assays, the therapeutic indices for amastigotes were 5.2 and 4.3 for polydatin and oxyresveratrol, respectively. The therapeutic index for pterostilbene was 5.4, and that for piceatannol was >8.9. Interestingly, although it has been reported that piceatannol causes a loss of mitochondrial potential and the release of cytochrome c [28], it was found to be the less toxic of the analogs tested with XTT, which measures mitochondrial dehydrogenases.

Duarte and colleagues [29] demonstrated that piceatannol has IC_50_ values of 4.2 μg/mL (17.2 μM), 3.9 μg/mL (16 μM) and 5.7 μg/mL (23.3 μM) for promastigotes of *L*. *donovani*, *L*. *infantum* and *L*. *major*, respectively. They also found an IC_50_ of 7.4 μg/mL (30.3 μM) for piceatannol against amastigotes of *L*. *donovani* in RAW macrophages. We determined that piceatannol is effective against *L*. *amazonensis* with an IC_50_ of 65 μM for promastigotes and an IC_50_ of 45 μM for intracellular amastigotes. Tolomeo and colleagues [30] showed that pterostilbene has LD_50_ values of 7.6 μg/mL (29.6 μM) and 10.3 μg/mL (40.2 μM) for the promastigote and amastigote forms of *L*. *infantum*. Corroborating these data, we found IC_50_ values of 17.7 μM and 33.2 μM for pterostilbene against *L*. *amazonensis* promastigotes and amastigotes, respectively.

To evaluate the leishmanicidal mechanisms activated by the *trans*-resveratrol analogs, we tested their ability to modulate NO production in murine macrophages. Our results demonstrated that pterostilbene and piceatannol decrease NO production by LPS-stimulated macrophages. Hsu *et al*. [31] showed that pterostilbene decreased the mRNA expression of iNOS in TNF-α-induced 3T3-L1 adipocytes. Moreover, resveratrol and pterostilbene inhibited the production of nitric oxide and the secretion of TNF-α by LPS-stimulated RAW 264.7 cells. Resveratrol and pterostilbene also inhibited the expression of TNF-α, IL-1β, IL-6, and iNOS genes in LPS-stimulated RAW 264.7 cells and LPS-stimulated peritoneal macrophages from both C57BL/6 and BALB/c mice [32]. Interestingly, we demonstrate that at least a portion of the NO inhibition by pterostilbene could be due to its capacity to scavenge NO, a property that it does not share with the other three analogs.

We showed that piceatannol reduces ROS production in agreement with Kim and colleagues [33], who demonstrated that 10 μM piceatannol attenuated hydrogen peroxide and peroxynitrite-induced apoptosis of a rat pheochromocytoma cell (PC12). Pterostilbene also decreased ROS in activated polymorphonuclear leukocytes [34]. It has been suggested that resveratrol and oxyresveratrol have protective effects against reactive oxygen and nitrogen species in a murine microglial cell line, by considerably decreasing NO and ROS levels [35]. Our results showed that oxyresveratrol decreased the production of ROS, but polydatin was a weak ROS inhibitor in unopsonized zymosan-stimulated murine macrophage Raw264.7 cells, human monocytes, and neutrophils in comparison to resveratrol [36]. In our assay conditions, piceatannol and oxyresveratrol were more potent ROS inhibitors than pterostilbene and polydatin. These data suggest that the leishmanicidal effect of the resveratrol analogs could not be attributed to ROS and NO generation in macrophages.

Previously, we showed that resveratrol increases promastigotes in the sub-G0/G1 phase of their cell cycle and that it induces mitochondrial membrane depolarization, suggesting that the parasites undergo incidental death [7]. Therefore, in the present study, the effect of *trans*-resveratrol analogs in the modulation of the parasite cell cycle was examined. Among the analogs tested, we found that only piceatannol affected the promastigote cell cycle, increasing the number of cells in the sub-G0 phase and decreasing the number of cells in the G0-G1 phase. Similarly to resveratrol, piceatannol changed the parasite mitochondrial membrane potential, suggesting incidental death as its killing mechanism. In agreement with our findings, Kita and colleagues [37] demonstrated that 25 and 50 μM piceatannol induced cell cycle arrest at the G2/M phase in AH109A hepatoma cells. Furthermore, piceatannol failed to induce apoptosis at 50 μM in AH109A hepatoma cells; however, at 100 μM, it induced strong apoptosis in those cells [37]. Another feature of the promastigote incidental death we observed was an increase in annexin-V binding to the surface of piceatannol-treated promastigotes.

The theoretical *in silico* pharmacokinetics approach is widely used in the initial study of ADMET (Absorption, Distribution, Metabolism, Excretion and Toxicity) properties (S1 Table) with the aim of reducing the costs of biological assays of compounds and a high probability of identifying future problems and toxicity; this saves both time and money [38]. It has been demonstrated *in vitro* that hepatic biotransformation of *trans*-resveratrol is dependent of the activity of *trans*-resveratrol isoform(s) and liver cytochrome P450 (CYP) [39]. The hepatic metabolism of *trans*-resveratrol into piceatannol and another tetrahydroxystilbenes has been shown to be catalyzed by recombinant human CYP1A1, CYP1A2 and CYP1B1. Our *in silico* ADMET properties validated that piceatannol may act allosterically to inhibit CYP1A2. We showed that resveratrol acts synergistically with amphotericin B against *L*. *amazonensis* [7]. Interestingly, CYP1A2 was not inhibited by amphotericin B; thus, it did not harm the metabolism of resveratrol (S1 Table).

The replacement of the hydroxyl group at carbon-3 with one on carbon-2 was not able to change the leishmanicidal effect of resveratrol as oxyresveratrol and piceatannol had similar IC_50_ values on promastigotes of *L*. *amazonensis*. However, their mechanisms of action are different; piceatannol alters the parasite cycle and oxyresveratrol the effect of is not clear. This highlights the importance of studying the correlations between structural changes and biological effects.

The results of our study show that *trans*-resveratrol analogs have anti-*Leishmania amazonensis* activity of *in vitro* and that piceatannol is capable of inducing promastigotes’ incidental death, suggesting that these compounds are promising candidates for future studies regarding leishmaniasis treatment.

## Supporting Information

S1 Table ADMET profile prediction of the *trans*-resveratrol analogs. (DOCX)
