# Supplementary material for: Leishmanicidal Effect of Synthetic trans-Resveratrol Analogs
Source: PLoS One. 2015 Oct 30;10(10):e0141778. doi: 10.1371/journal.pone.0141778 (PMC4627731; doi:10.1371/journal.pone.0141778)
Supplement: S1 Table — (DOCX) [file pone.0141778.s001.docx]

|  |  | **PTEROSTILBENE** | | **PICEATANNOL** | | **POLYDATIN** | | **OXYRESVERATROL** | | **AMB** | |
| --- | --- | --- | --- | --- | --- | --- | --- | --- | --- | --- | --- |
|  |  | R | P | R | P | R | P | R | P | R | P |
| **ABSORPTION** | Blood-Brain Barrier | + | 0.8164 | - | 0.5495 | + | 0.6876 | + | 0.7206 | - | 0.9748 |
|  | Human Intestinal Absorption | + | 0.9974 | + | 0.9526 | - | 0.5481 | + | 0.9829 | - | 0.9180 |
|  | Caco-2 Permeability | + | 0.8911 | + | 0.5789 | - | 0.8319 | + | 0.8545 | - | 0.7006 |
|  | P-glycoprotein Substrate | Non-substrate | 0.6184 | Non-substrate | 0.5669 | Substrate | 0.5330 | Non-substrate | 0.6334 | Substrate | 0.8691 |
|  | P-glycoprotein Inhibitor | Non-inhibitor | 0.7001 | Non-inhibitor | 0.9237 | Non-inhibitor | 0.7722 | Non-inhibitor | 0.9231 | Non-inhibitor | 0.5173 |
|  |  | Non-inhibitor | 0.7942 | Non-inhibitor | 0.9761 | Non-inhibitor | 0.8607 | Non-inhibitor | 0.9704 | Inhibitor | 0.5498 |
|  | Renal Organic Cation Transporter | Non-inhibitor | 0.8259 | Non-inhibitor | 0.9072 | Non-inhibitor | 0.8380 | Non-inhibitor | 0.9768 | Non-inhibitor | 0.8507 |
| **METABOLISM** | CYP450 2C9 Substrate | Non-substrate | 0.7415 | Non-substrate | 0.7658 | Non-substrate | 0.7823 | Non-substrate | 0.7409 | Non-substrate | 0.8987 |
|  | CYP450 2D6 Substrate | Non-substrate | 0.8923 | Non-substrate | 0.9068 | Non-substrate | 0.8868 | Non-substrate | 0.9073 | Non-substrate | 0.8158 |
|  | CYP450 3A4 Substrate | Non-substrate | 0.5993 | Non-substrate | 0.6661 | Non-substrate | 0.6124 | Non-substrate | 0.6847 | Substrate | 0.5478 |
|  | CYP450 IA2 Inhibitor | Inhibitor | 0.8736 | Inhibitor | 0.9107 | Non-inhibitor | 0.9329 | Inhibitor | 0.9515 | Non-inhibitor | 0.8600 |
|  | CYP450 2C9 Inhibitor | Non-inhibitor | 0.9304 | Inhibitor | 0.6182 | Non-inhibitor | 0.8927 | Inhibitor | 0.9190 | Non-inhibitor | 0.8614 |
|  | CYP450 2D6 Inhibitor | Non-inhibitor | 0.9249 | Non-inhibitor | 0.9231 | Non-inhibitor | 0.9183 | Non-inhibitor | 0.9195 | Non-inhibitor | 0.8518 |
|  | CYP450 2C19 Inhibitor | Inhibitor | 0.7583 | Non-inhibitor | 0.9026 | Non-inhibitor | 0.8555 | Inhibitor | 0.8449 | Non-inhibitor | 0.8149 |
|  | CYP450 3A4 Inhibitor | Non-inhibitor | 0.5979 | Inhibitor | 0.5427 | Non-inhibitor | 0.8716 | Inhibitor | 0.7134 | Inhibitor | 0.6075 |
|  | CYP Inhibitory Promiscuity | High | 0.6700 | High | 0.7651 | High | 0.5977 | High | 0.9090 | Low | 0.9781 |
| **TOXICITY** | AMES Toxicity | Non-toxic | 0.8557 | Non-toxic | 0.6502 | Non-toxic | 0.5661 | Non-toxic | 0.9172 | Non-toxic | 0.8136 |
|  | Carcinogens | Non-carcinogens | 0.7521 | Non-carcinogens | 0.8962 | Non-carcinogens | 0.9556 | Non-carcinogens | 0.8521 | Non-carcinogens | 0.9374 |
|  | Acute Oral Toxicity | III | 0.6558 | III | 0.7909 | III | 0.6904 | III | 0.7754 | III | 0.6621 |

R - results, P - probability.

*In silico* ADMET theoretical analyses were carried out as proposed by Pinheiro et al. (24), and different descriptors shown were evaluated using admetSAR (http://lmmd.ecust.edu.cn:8000) methods.
